# Perceived benefits and constraints of artificial intelligence integration in teachers’ college libraries: a TAM-based study

**DOI:** 10.3389/frai.2026.1751832

**Published:** 2026-05-21

**Authors:** Inos Chibidi, Logic Magwa

**Affiliations:** 1 Naveen Jindal School of Management, The University of Texas at Dallas, Richardson, TX, United States; 2 College of Education, Institute for Open Distance Learning, University of South Africa, Pretoria, South Africa

**Keywords:** artificial intelligence, constraints, integration, library and information services, machine learning, perceived benefits, TAM-based study

## Abstract

The Fourth Industrial Revolution (4IR) has accelerated the adoption of Artificial Intelligence (AI) across knowledge intensive sectors, yet its integration in Library and Information Services (LIS) within resource constrained educational environments remains limited and under examined. This empirical paper seeks to explore the impact of AI integration in LIS and the associated challenges. This research examines the various applications of AI in libraries. The paper intends to identify the benefits and challenges associated with AI integration in libraries. This study adopted a qualitative descriptive design informed by phenomenological principles to study how AI application is perceived by key stakeholders to be beneficial and what barriers constrain AI uptake, and in what new areas AI can be applied in teachers’ college libraries. The study was anchored to the Technology Acceptance Model (TAM) where the effect of Perceived Usefulness (PU) and Perceived Ease of Use (PEOU) on the stakeholders’ preparedness to adopt AI was analysed as an interpretive lens. Sixty participants (library staff, ICT experts and Computer Science students) were interviewed in-depth, participated in focus group discussions, and were observed using structured observations. All data was analysed thematically using Braun and Clarke’s six-step framework. Evidence found that there was no real implementation of AI in academic libraries. Actual usage was almost non-existent, mainly automated processes within existing library systems, such as those integrated within Koha. Nonetheless, the participants perceived AI as potentially useful for automating mundane tasks, increasing ease in locating information, providing an improved user experience, and for continuous availability through the use of chatbots. In spite of the strong perceived usefulness, the actual PEOU and institutional preparedness for AI are limited by infrastructure challenges, lack of funds, limited digital literacies of users, ethical issues, and wider issues of digital disparity. The study stresses the need for a context-specific AI governance strategy, building capacity of all stakeholders, and adoption of a phased approach in low-resource academic libraries. The findings provide new insights into Global South literature by presenting an in-depth, multi-stakeholder view of teachers’ college AI readiness, emphasizing the interplay between technological novelty, infrastructural challenges, and ethics.

## Introduction and background of the study

The 4th Industrial Revolution (4IR), characterized by digitization and digitalization, continues to reshape how information is created, organized, accessed, and distributed across all levels of society (Moyo, 2022). Libraries and Information Services (LIS), traditionally pillars of knowledge management and organization, are at the forefront of this technological era. In this 4IR era, information has increasingly been converted into digital format (digitalization). The sudden growth in Big Data, which has seen large volumes of varied data being shared at tremendous velocities, has also promoted the integration of Artificial intelligence (AI), defined as the simulation of human intelligence processes by machines (Heath, 2018) in information management institutions like libraries. Libraries are therefore supposed to transform their operations to be in line with the needs and requirements of the current information age. To remain relevant, libraries need to leverage the transformative power of Artificial Intelligence in their service provisions to clients.

AI, which encompasses areas such as machine learning (ML), natural language processing (NLP), and intelligent automation (Heath, 2018), is emerging as a powerful tool capable of revolutionizing fundamental library functions, including cataloguing, classification, information retrieval, user assistance, and management of digital resources (Cox et al., 2019; Luo and Qin, 2021). Like other fields, libraries are not spared by the transformative power of AI (Das and Islam, 2022). AI has recently entered into a boom with the advent of such technologies as Machine Learning (ML) and Deep Learning (DL) (Miyazaki et al., 2018 cited in Kopka and Fornahl, 2023). The use of Machine Learning tools has made it easier to apply AI to many different applications and fields, packaging it as a one-size-fits-all solution to several issues. Academic libraries worldwide are experimenting with AI-enabled tools, ranging from recommender systems and automated metadata generation to chatbots and predictive analytics, reflecting a widespread trend toward data-driven service models (Fernandez, 2023; Yoon, 2022).

Despite this accelerating global trend, the implementation of AI in libraries remains inconsistent, especially in resource-constrained settings. Teachers’ colleges in many developing nations grapple with persistent challenges such as limited funding, inadequate digital infrastructure, unreliable connectivity, and a shortage of skilled professionals (Onyancha, 2022; Musakali and Mutula, 2023). These systemic constraints significantly influence the feasibility of AI integration as well as the expectations and readiness of library staff and users alike. As global AI governance frameworks—such as the Recommendation on the Ethics of Artificial Intelligence by United Nations Educational, Scientific and Cultural Organization (UNESCO) (2021), the AI Principles of the OECD (2019), and the Continental AI Strategy of the African Union (2024) highlight the importance of equitable and contextually relevant adoption, a deeper understanding of how AI is perceived and negotiated within low-resource educational contexts is crucial (Chen et al., 2020).

The field of information access and management is going through a significant change inspired by the booming fields of AI and ML. AI integration has proved to be the best solution to the information processing and sharing needs of library patrons. It is therefore necessary to find out the constraints and benefits in the integration of AI in information management institutions like libraries. This paper seeks to explore the various applications of AI as they are being integrated into LIS, exploring their potential to revolutionize the way information is categorized, accessed, and utilized while clearly highlighting the constraints and challenges. The potential of AI to transform library operations has been broadly outlined (Omame and Alex‑Nmecha, 2020).

## Literature review

Artificial Intelligence (AI) has become a pivotal area of research in Library and Information Services (LIS) due to its potential to automate various processes, manage vast amounts of data, and enhance user services. As a result, academic libraries globally are increasingly exploring the use of AI-enabled tools to improve efficiency, user satisfaction, and data-driven decision-making. This literature review consolidates significant research on AI applications in libraries, emerging opportunities, and existing limitations, with a focus on relevance to resource-constrained environments like Zimbabwean teachers’ colleges.

### AI applications in library and information services

Various researches have been conducted on the integration of artificial intelligence in different organizations and institutions. Brynjolfsson et al. (2019) as cited in Okunlaya et al. (2022) conducted a research study on how useful AI is to business organizations. The study concluded that almost all industries were transforming their fundamental processes and business models in order to benefit from AI. As early as 1990, Riddick speculated on the role of AI in supporting reference librarians.

According to Al-Shihi et al. (2018), artificial intelligence has also recently revolutionized the library sector. The researcher noted that libraries, being the very important information centers for academic institutions, were not spared by the disruptive nature of AI. The researcher argued that the current digital era has necessitated libraries to adopt AI in their various operations in order to remain relevant to their clients. Universities are currently changing their teaching, learning and research models to adapt to the changes that are being brought by the disruptive nature of AI (Al-Shihi et al., 2018).

A growing range of AI applications in library operations is highlighted in recent scholarship. Systematic reviews by Ali et al. (2021) indicate that AI is being utilized for automated cataloguing, classification, metadata extraction, information retrieval, and digital preservation. These studies suggest that the accuracy and speed of bibliographic control have been improved through machine learning and natural language processing (NLP), thereby reducing manual effort and enhancing resource discoverability.

Das and Islam (2022) also carried out research in India to identify the application of artificial intelligence and machine learning in libraries, while assessing how these technologies could assist and support the library operations and services. The researchers found out that the current state of the AI and ML that was relevant within the LIS domain mainly focused on theoretical work and much was to be done on implementation. The researchers also found out that library operations like cataloguing, classification, indexing, document analysis and text recognition were major areas of AI application in libraries. The researchers also discovered that AI was used for metadata generation, resource discovery and book acquisition. The researchers concluded that in order for libraries to benefit from AI integration, much needs to be done on the practical implementation of the integration process.

Cox et al. (2019) propose that AI is transforming the “intelligent library” by incorporating predictive analytics, recommender systems, and automated workflows to support personalized user services. Similarly, Zhang and Zhao (2023) discuss advances in AI-driven information retrieval, noting that deep learning models now outperform traditional search engines in relevance ranking and semantic understanding.

Another research worth mention was carried out by Wu et al. (2015) as cited in Subaveerapandiyan (2023), on application of artificial intelligence (AI) and Machine learning (ML) technologies in CiteSeerX which is a digital library search engine. The research discovered key AI and ML tools for document classification, de-duplication and metadata extraction. The research emphasised on the unique features of CiteSeerX, including open access to full-text scholarly documents, automatic extraction of metadata and citation context. The research discussed the benefits of AI and ML technologies including their transferability to other digital libraries.

AI-powered chatbots and virtual assistants have also become increasingly popular. From a similar perspective, Thalaya and Puritat (2022); (Rubin et al., 2010) carried out the BCNPYLIB CHAT BOT experiment in Thailand to find out how AI and ML technologies can be applied in libraries to improve library services and increase user satisfaction. User acceptance of AI‑powered shopping assistants has been studied in e‑commerce (Araújo and Casais, 2020), and similar acceptance factors apply to library chatbots. The trial results from the experiment showed that employing AI and ML tools to respond to user queries about book locations, opening times, and other pertinent information helped to save the librarian’s time and enhanced the management of library operations and services. The satisfaction survey findings revealed an increase of 0.45% among nursing students and instructors, which improved library service management. Yoon (2022) and Fernandez (2023) also document the adoption of conversational agents by academic libraries to offer 24/7 reference support, respond to frequent inquiries, and guide users through digital platforms. These tools improve access to library services and reduce the workload on human librarians, particularly during periods of high demand.

On another note, Massis (2018) also carried out research in Columbus State Community College in the United States of America to examine artificial intelligence (AI) and its potential relationship to the library. The research discovered potential implications of AI in libraries which were categorized as both exciting and disruptive. The research however unearthed some concerns that AI technologies could disrupt the library’s operations and services. The author also acknowledged that acceptance and incorporation of AI technologies in libraries positively enhanced library services.

In support of this, Ali et al. (2021) carried out research in Pakistani to highlight and discuss the effects of AI on library operations and user services. The researchers found out that the adoption of AI in libraries has resulted in fast, accurate and reliable services which promote library efficiency. The researchers also found out cataloguing, classification and recommender systems as major application areas of AI in libraries in Pakistani. The researchers discovered that AI is still a new technology in Pakistani and most of the universities in Pakistani were using Koha open-source software which has some limited AI functionality embedded in it. The researchers concluded that majority of Pakistani libraries are using AI to support the Radio-Frequency Identification (RFID) technology for library security, stock management as well as the check-in check-out process.

With regards to application areas for AI in libraries, Asemi and Asemi (2018) explored the potential for library systems to use AI techniques and the application of artificial intelligence (AI) in Iran’s library systems. The research was meant to unearth the various application areas of AI and ML in the library. The researchers found out that, of the various application areas of AI and ML in the library, natural language processing was the least used, while recommender systems were the most used application. Beyond AI, blockchain technology is also being explored for library services (Jha, 2023), though its adoption faces similar infrastructural barriers. In addition, cloud computing, defined by Mell and Grance (2009), provides a foundational infrastructure that can support AI deployment in low‑resource libraries.

### AI adoption in developing country and global south contexts

While AI adoption is accelerating worldwide, its implementation in developing nations remains uneven. Studies from Africa and Asia identify infrastructural limitations, funding constraints, and skill shortages as significant factors affecting the feasibility of AI integration (Musakali and Mutula, 2023; Onyancha, 2022). Research from Pakistan, India, and Iran reveals that libraries often utilize open-source systems like Koha, which offer limited AI functionalities (Ali et al., 2021; Asemi and Asemi, 2018). Studies from Nigeria report positive perceptions of AI’s impact on academic library services (Ogwo et al., 2023), aligning with the aspirational PU found in our study. Such contexts closely resemble the situation in Zimbabwe, where AI adoption is emerging but largely theoretical.

From the Zimbabwean context, Mupaikwa (2024) carried out a research on “The application of Artificial Intelligence and Machine Learning in Academic Libraries in Zimbabwe.” The researcher found out that the application of AI and ML in libraries has spread across all key sections of the library and have helped with strategic decision-making. Mupaikwa (2024) explained that AI and ML use has provided unlimited opportunities for the reduction of operational costs in a library, allowing the reassignment of library staff to other critical areas of the library. The researcher also noted that there has been limited adoption of AI and ML due to several challenges including technological, organisational, human as well as environmental. Furthermore, the researcher noted that despite the challenges faced in AI and ML adoption in libraries, the use of AI and ML have improved library services and operations. The research also revealed that AI and ML adoption demanded new skills among library professionals. The researcher suggested the need to train library staff to acquaint them with skills required to operate with AI based tools.

Scholarly research from the Global South also emphasizes the importance of context-specific AI adoption. Luo and Qin (2021) argue that an academic library’s readiness for AI depends not only on its technological infrastructure but also on institutional culture, digital literacy, and governance structures. This aligns with research from African LIS that highlights the role of existing digital divides, unreliable connectivity, and limited opportunities for professional development as barriers to AI adoption (Musakali and Mutula, 2023).

### Ethical, governance, and digital inequality considerations

The adoption of AI in libraries raises significant ethical and governance concerns. Global guidelines such as UNESCO’s Recommendation on the Ethics of Artificial Intelligence [United Nations Educational, Scientific and Cultural Organization (UNESCO), 2021] and the OECD AI Principles (OECD, 2019), and the House of Lords Select Committee on Artificial Intelligence (2018) stress the need for transparency, accountability, fairness, and human oversight. These principles are particularly relevant in regions where algorithmic bias, data privacy issues, and surveillance risks may disproportionately affect marginalized communities.

Critics like Noble (2018) and Eubanks (2018) warn that AI systems can perpetuate existing inequalities if not implemented with appropriate safeguards. Their work underscores the importance of responsible AI adoption in libraries, especially in resource-constrained environments, to avoid exacerbating digital exclusion or undermining user rights.

Digital inequality theory is a useful lens for understanding AI adoption in low-resource contexts. Warschauer (2004) and others argue that technology access is shaped by social, economic, and infrastructural factors. In Zimbabwean teachers’ colleges, limited bandwidth, outdated hardware, and insufficient training influence perceived ease of use (PEOU) and perceived usefulness (PU), the key constructs in the Technology Acceptance Model (TAM).

### Gaps in existing literature

The current research on “Artificial Intelligence integration in Zimbabwean Library and Information Services” is different from the previous research in terms of context and setting. The previous research was carried out in different contexts from the Zimbabwean context under study. Most of the findings obtained in previous research cannot be easily transferred to the Zimbabwean context, hence this current research is worth carrying out.

Despite growing interest in AI in LIS, several gaps remain in current research:

1. Limited empirical studies in African teacher training institutions: most studies focus on University libraries, neglecting teachers’ colleges, which are institutions that play a critical role in shaping digital literacy and pedagogical innovation.
2. Overemphasis on technical potential versus lived experience: much of the literature is conceptual or technical, with few studies exploring perceptions, readiness, and contextual constraints in settings where AI adoption is still nascent.
3. Insufficient integration of governance and digital inequality frameworks: few LIS studies critically engage with global AI ethics or structural barriers in developing countries.
4. Lack of cross-stakeholder perspectives: existing research rarely includes librarians, ICT experts, and students within the same institutional setting.

The research by Mupaikwa (2024) in Zimbabwe is generalized across all academic libraries in Zimbabwe. It is different from the current study which seeks to find benefits and constraints of AI integration in LIS in teachers’ colleges in Masvingo Province. The current study seeks to find out how far teachers’ colleges in Masvingo have gone in integrating AI in their library and Information Services and to determine the impact of these technologies on operations and services offered to library patrons. It also seeks to find out the benefits and challenges associated with AI integration in libraries.

This study addresses these gaps by providing a multi-stakeholder, context-specific analysis of AI integration in teachers’ college libraries in Masvingo Province, Zimbabwe. It contributes to Global South scholarship on AI in LIS by exploring early-stage perceptions, identifying opportunities and constraints, and situating these findings within broader socio-technical and governance contexts.

This paper is organized as follows: theoretical framework, statement of the problem, research methodology, findings, discussion, conclusion, and recommendations.

### Theoretical framework

The study was underpinned by the Technology Acceptance Model (TAM) that was propounded by Davis (1989). The model explains the acceptance of new technologies and information systems by individuals. TAM postulates that the acceptance of technology and information systems is predicted by the users’ behavioural intention, which in turn is determined by the perception of technology’s usefulness in performing the task and perceived ease of its use (Marikyan and Papagiannidis, 2024).

The major goal of TAM was to shed light on the processes that influence the acceptance of technology and information systems. This helps to predict the behaviour of individuals and provide a theoretical explanation for the successful implementation of the said technology. TAM was also used to inform practitioners about measures that they must take prior to the implementation of information systems and new technologies. TAM can be diagrammatically illustrated as shown below.

When new technology is introduced, the acceptance and use of the new technology is determined by two external variables; namely *perceived usefulness* and *perceived ease of use*.

TAM suggested that an individual’s decision to perform a certain behaviour is mostly the result of the analysis of the benefit that are expected from the behaviour compared to the effort or costs incurred to perform the behaviour (Johnson and Payne, 1985; Payne, 1982). This points to the fact that the use of the information system is determined by an evaluation of the trade-off between the perceived usefulness of the new system or technology and the perceived difficulty of using it (Davis, 1989).

### Perceived usefulness (PU)

Perceived usefulness was defined as the individual’s perception of the extent to which the use of a given new technology improves performance. TAM model aligns very well with Bandura’s concept of outcome judgement, which talks about an individual’s expectation of a positive outcome triggering his or her behaviour (Bandura, 1982). When library staff deem AI useful in their operations, they will have more chances of integrating it into the Library information services.

### Perceived ease of use (PEOU)

Perceived ease of use was defined as the extent to which a person believes that using a particular technology or system requires less effort (Davis, 1989). The theory derived from the self-efficacy concept, which refers to a situation-specific belief about how easy the individual finds it to execute a task (Davis, 1989; Bandura, 1982). In this study, library patrons and staff may find it easy to integrate AI when it is easy for them to use the technology.

### Intention to use

The intention to use the new technology and systems is directly impacted by the users’ perception of how useful the technology is as well as how easy it is for users to maneuver the new technology (Marikyan and Papagiannidis, 2024). When users find the new technology very useful and if they have the requisite skills required to use the new technology with ease, their intention to use the technology increases. High intentions to use translate to actual use. There is a strong correlation between intention to use and the actual use.

### Relevance of TAM to AI integration in libraries

This research used the Technology Acceptance Model (TAM) to examine the factors influencing librarians’ and patrons’ acceptance of AI-powered services within the library environment. This theory is relevant in this study since TAM has been extensively used and validated across diverse technological domains, including information systems, e-commerce, and education. TAM’s proven track record lends credibility to its application in the library context. The researcher also chose TAM because it simplifies the complex process of adoption of technology by focusing on two key beliefs: perceived usefulness (PU) and perceived ease of use (PEOU). These beliefs are key drivers of user acceptance of new technology like artificial intelligence.

In the field of Library and Information Services (LIS), TAM offers a valuable analytical framework for examining how librarians, ICT staff, and students perceive emerging AI tools. Studies show that PU in the library context is often linked to expectations of improved efficiency, automation of routine tasks, enhanced information retrieval, and personalized user services (Ali et al., 2021; Luo and Qin, 2021). PEOU, on the other hand, is influenced by users’ digital literacy, training, system design, and the availability of technical support. These constructions are particularly relevant in academic libraries, where staff and users must navigate new technologies while managing existing workloads and infrastructural limitations.

This study utilizes TAM not as a quantitative predictive model but as an interpretive analytical framework to structure the thematic analysis. Participants’ descriptions of potential benefits (e.g., automation, improved access, enhanced user experience) are interpreted as manifestations of PU, while concerns related to skills shortages, inadequate infrastructure, and system complexity are viewed as factors affecting PEOU. This qualitative approach to applying TAM aligns with research traditions that use theoretical models to guide meaning-making.

### Critiques and limitations of TAM in low-resource contexts

Despite its widespread use, TAM has been criticized for its limitations, particularly in contexts where structural and environmental factors significantly shape technology adoption. TAM assumes that technology adoption decisions are primarily individual, whereas in low-resource settings, institutional capacity, infrastructure, funding, and policy play crucial roles in adoption (Cox et al., 2019; Musakali and Mutula, 2023). These structural elements fall outside the original scope of TAM.

Moreover, TAM does not explicitly account for digital inequality, which is central to understanding technology adoption in developing countries. Digital inequality theory suggests that access to technology is shaped by social, economic, and infrastructural disparities (Warschauer, 2004). In Zimbabwean teachers’ colleges, issues such as limited bandwidth, outdated hardware, and inconsistent electricity supply directly impact PEOU and PU, but these are not addressed by TAM’s internal belief constructs.

TAM also does not incorporate AI governance and ethical considerations, which are increasingly important. Global frameworks like the UNESCO Recommendation on the Ethics of AI [United Nations Educational, Scientific and Cultural Organization (UNESCO), 2021] and the OECD AI Principles (OECD, 2019) emphasize transparency, accountability, fairness, and human oversight, the factors that affect institutional readiness but are outside TAM’s initial formulation.

### Integrating TAM with broader socio-technical perspectives

To overcome these limitations, this study complements TAM by incorporating insights from:

- Digital inequality theory (Noble, 2018; Eubanks, 2018; Warschauer, 2004)
- AI governance frameworks [United Nations Educational, Scientific and Cultural Organization (UNESCO), 2021; OECD, 2019]
- Institutional readiness models (Luo and Qin, 2021)

This integrated approach allows for a more comprehensive analysis of AI acceptance in resource-constrained academic libraries. It acknowledges that PU and PEOU are influenced not only by individual perceptions but also by infrastructure quality, availability of training, institutional policies, ethical concerns and broader socio-economic contexts. By situating TAM within these wider socio-technical realities, the study offers a nuanced understanding of AI readiness in Zimbabwean teachers’ colleges.

### Operationalization of TAM in this study

To ensure that TAM was used effectively, not merely as a decorative element, its constructs were explicitly operationalized:

- Interview and FGD questions were designed to elicit perceptions related to PU and PEOU.
- Statements made by participants were coded and mapped to TAM constructs.
- Themes
- were analyzed through the lens of PU (benefits) and PEOU (constraints).
- Cross-stakeholder comparisons were made to highlight variations in perceptions across librarians, ICT experts, and students.

This approach ensured that TAM guided the analytical process while remaining responsive to the contextual specificities of the study.

### Statement of the problem

Artificial Intelligence (AI) is increasingly recognized as a transformative force in Library and Information Services (LIS), with global research demonstrating its potential to automate routine tasks, enhance information retrieval, personalize user services, and improve operational efficiency (Ali et al., 2021; Cox et al., 2019; Zhang and Zhao, 2023). AI and ML integration in libraries has proved to be beneficial to all library stakeholders including library staff and patrons.

Despite these documented benefits, AI adoption remains uneven across regions, particularly in developing countries where infrastructural, financial, and skills-related constraints significantly shape technological readiness (Musakali and Mutula, 2023; Onyancha, 2022). In Zimbabwe, empirical research on AI integration in academic libraries is limited. Existing studies tend to provide broad national overviews rather than institution-specific analyses, and they rarely examine teachers’ colleges, institutions that play a critical role in shaping digital literacy and pedagogical innovation (Mupaikwa, 2024). As a result, little is known about how AI is currently understood, experienced, or anticipated within teachers’ college libraries, where adoption remains minimal and emergent.

Furthermore, global AI governance frameworks such as UNESCO’s Recommendation on the Ethics of AI [United Nations Educational, Scientific and Cultural Organization (UNESCO), 2021] and the OECD AI Principles (OECD, 2019) emphasize the need for equitable, context-sensitive adoption. However, the realities of digital inequality including limited bandwidth, outdated infrastructure, and insufficient professional training continue to constrain the perceived usefulness (PU) and perceived ease of use (PEOU) of AI based tools in many Global South contexts (Noble, 2018; Eubanks, 2018; Warschauer, 2004). These structural barriers fall outside the scope of traditional technology acceptance models and require contextually grounded investigation.

Given these gaps, there is a pressing need to understand how librarians, ICT experts, and technologically literate students in Zimbabwean teachers’ colleges perceive the benefits, constraints, and potential application areas of AI in LIS. Such insights are essential for informing institutional planning, capacity building, and policy development aimed at enabling responsible and meaningful AI adoption in resource-constrained academic environments.

It is against this background that the current study sought to find out the benefits and constraints in AI and ML integration in Zimbabwean libraries. The study also sought to find out the various application areas of AI and ML in Library and Information Services in the Zimbabwean context. The current study was guided by the following main research question and sub questions:

To address the identified problem, the study was guided by the following overarching research question and sub-questions:

### Main research question

What are the perceived benefits and constraints of Artificial Intelligence (AI) integration in Library and Information Services in teachers’ colleges in Masvingo Province, Zimbabwe?

### Sub-research questions

1. What is the current state of AI integration in Library and Information Services in teachers’ colleges in Masvingo Province?
2. What are the perceived application areas of AI in Library and Information Services within these institutions?
3. What constraints and challenges affect the perceived ease of use (PEOU) and feasibility of AI integration in these libraries?

## Research methodology

### Research design

This study adopted a qualitative descriptive design informed by phenomenological principles. While phenomenology traditionally seeks to uncover the essence of lived experience, this study did not pursue full phenomenological reduction or interpretive phenomenological analysis. Instead, phenomenological sensitivity was used to explore how participants perceive, experience, and make meaning of emerging AI integration in Library and Information Services (LIS).

A qualitative descriptive approach was selected because AI adoption in the studied libraries is minimal and emergent, making it necessary to capture participants’ accounts in their own words without imposing a highly abstract interpretive framework. This design is appropriate for studies aiming to provide a rich, contextualized description of a phenomenon as experienced by multiple stakeholder groups (Sandelowski, 2000; Bradshaw et al., 2017).

The approach aligns with the study’s exploratory purpose and supports the use of the Technology Acceptance Model (TAM) as an interpretive lens rather than a predictive model. TAM constructs, which include Perceived Usefulness (PU) and Perceived Ease of Use (PEOU) guided the development of interview questions and informed the thematic interpretation of participants’ perceptions.

### Study setting

The study was conducted in three teachers’ colleges located in Masvingo Province, Zimbabwe. These institutions were selected because they:

1. Operate functional academic libraries
2. Are situated in a region accessible to the researcher
3. Represent typical resource-constrained educational environments in the Global South

Teachers’ colleges play a critical role in shaping digital literacy and pedagogical innovation, yet they remain under-represented in AI-in-LIS research.

### Sampling strategy

A purposive sampling strategy was used to select information-rich cases and participants. Three colleges (referred to as College A, B, and C) were selected based on relevance to the research problem. Within each college, participants were purposively selected from three stakeholder groups:

1 Library staff (*n* = 6)

Two staff members per college (one professional librarian and one library assistant). They provided insights into operational workflows, service delivery, and practical challenges.

2 ICT experts (*n* = 6)

Two ICT personnel per college. They contributed technical perspectives on infrastructure, system capabilities, and feasibility of AI integration.

3 Computer science students (*n* = 48)

Sixteen final-year students per college. They were included as technologically literate informants capable of evaluating digital systems and identifying potential AI applications (see Tables 1, 2).

**Table 1:** Research participants.

Category	Number (*n*)	Research site	Inclusion criteria
1. Library Staff	6	College Library	Male and female people with knowledge of daily operations in the library
2. IT experts	6	College	Male and Female experts in ICT technologies
3. Computer science students	48	College	Male and Female students with an appreciation of ICT technologies

**Table 2:** Sample distribution across colleges.

Case	Library staff	ICT experts	CS students	Total
College A	2	2	16	20
College B	2	2	16	20
College C	2	2	16	20
Total	6	6	48	60

This multi-stakeholder sampling enabled data triangulation and a holistic understanding of AI readiness.

### Data collection methods

Three complementary methods were used:

#### In-depth interviews

In-depth interviews were conducted with library staff and ICT experts. Interviews explored current digital systems, perceptions of AI usefulness (PU), perceived ease of use (PEOU), institutional readiness and constraints and ethical concerns. Interviews lasted 45–60 min and were audio-recorded with consent.

#### Focus group discussions (FGDs)

Three FGDs were conducted with Computer Science students (one per college). Each FGD lasted approximately 60–90 min and explored awareness of AI tools, perceived potential applications, user experience with existing systems as well as expectations and concerns of users.

FGDs enabled collective meaning-making and peer-supported reflection.

#### Participant observation

The researcher conducted 3-h observation sessions per week for 3 weeks in each library, totaling 27 h of observation across all three institutions. Observations focused on:

- Library workflows
- User–librarian interactions
- System usage patterns
- Evidence of automation or digital constraints
- Infrastructure quality and connectivity

An observation checklist ensured consistency across sites.

### Data analysis

Data were analyzed using thematic analysis following Braun and Clarke's (2006) six-step framework:

1. Familiarization: transcripts were read repeatedly to gain an overall understanding.
2. Initial coding: codes were generated inductively from the data and deductively using TAM constructs (PU and PEOU).
3. Theme development: codes were grouped into broader themes reflecting benefits, constraints, current state, and application areas.
4. Theme review: themes were refined through peer debriefing and cross-checking with raw data.
5. Theme definition: themes were clearly defined and linked to TAM, digital inequality theory, and AI governance frameworks.
6. Reporting: findings were presented with illustrative quotes and cross-stakeholder comparisons.

### Trustworthiness and rigor

To enhance credibility, dependability, confirmability, and transferability, the study employed:

- Data triangulation through the use of different instruments (interviews, FGDs, observations)
- Prolonged engagement in the field (three weeks per institution)
- Peer debriefing with academic colleagues
- Thick description of context and participants
- Reflexive journaling to minimize researcher bias

These strategies align with qualitative research standards (Lincoln and Guba, 1985).

### Reflexivity statement

The researcher acknowledges that their background in ICT and LIS may influence interpretations of AI readiness. To mitigate bias:

- Reflexive notes were kept throughout data collection and analysis
- Interpretations were cross-checked with peers
- Participants’ voices were prioritized through verbatim quotations
- Theoretical assumptions were explicitly stated

This reflexive stance strengthened the transparency and credibility of the study.

### Data presentation and analysis

It is important to note that the findings presented here reflect perceived, emerging, and limited applications of AI within the libraries studied. None of the three teachers’ colleges had fully implemented AI systems; instead, AI functionalities were largely embedded within existing library management systems such as Koha. The findings therefore illuminate early-stage perceptions, institutional readiness, and socio-technical constraints shaping AI adoption in resource-constrained academic environments.

Four major themes emerged from the data:

1. Current state of AI integration in library and information services
2. Perceived benefits of AI Integration in LIS
3. Constraints and challenges affecting AI adoption
4. Perceived application areas for AI in LIS

These themes are presented below with cross-stakeholder comparisons and interpretive links to TAM constructs.

## Theme 1: Current state of AI integration in library and information services

Across all three colleges, participants consistently described AI integration as minimal, emergent, and largely theoretical. Library staff reported that their current systems, primarily Koha, contained limited automated features such as basic search algorithms, automated overdue notices, and rudimentary cataloguing support. However, these were not recognized as “AI” by most participants.

A librarian from college A noted:

*"We do not have AI systems as such. What we have are basic automated functions in Koha, but nothing advanced like chatbots or intelligent search tools."*

Supporting the same idea, the ICT expert during the interview session said:

*"The current state of AI-based technologies in teachers colleges libraries is still pathetic. We are using the least of these technologies. Currently the AI that we are using is embedded in the Koha library open source systems that we are using. But there is room to explore more if we fully integrate AI in our libraries."*

ICT experts confirmed this assessment, emphasizing that infrastructural limitations and outdated hardware constrained the adoption of more advanced AI tools. Students similarly reported that they had never interacted with AI-enabled library services, though many expressed awareness of AI applications in other domains.

Responding to the question on current state of AI and ML integration in teachers colleges libraries, one participant from Focus Group Discussion 1 had this to say:

*"We have read about AI and ML technologies and have heard about their benefits but we haven't seen much in our libraries that suggest their integration."*

A Focus Group Discussion 2 participant responded to the question on current state of AI and ML integration in libraries by stating that:

*"AI and ML technologies have not been adequately integrated in libraries. We have heard about these technologies but their integration in libraries is not yet evident because we are still experiencing the same type of services."*

In the same vein, a participant from Focus Group Discussion 3 reiterated that:

*"AI-based technologies are still new in libraries and have not been adequately integrated. A lot needs to be done if we are to enjoy the benefits of AI integration in libraries."*

When the researcher observed the library systems, services and operations, it was noted that AI integration is still very low in teachers’ college libraries. Those Colleges with some AI technologies were those using the Koha system which is an open-source software suite.

The sentiments of the research participants on the current state of AI and ML integration in libraries concurred with the findings by Das and Islam (2022) who carried out research in India on “Application of Artificial Intelligence and Machine Learning in Libraries.” The researchers sought to identify the application of artificial intelligence and machine learning in libraries, while assessing how these technologies could assist and support the library operations and services. The researchers found out that the current state of the AI and ML that was relevant within the LIS domain mainly focused on theoretical work and much was to be done on implementation. This view was also supported by Ali et al. (2021) who carried out a research in Pakistani on “Artificial Intelligence (AI) in Pakistani University library services.” The main objective of the study was to highlight and discuss the effects of AI on library operations and user services. The researchers discovered that AI is still a new technology in Pakistani and most of the universities in Pakistani were using Koha open-source software which has some limited AI functionality embedded in it.

### Interpretation through TAM

- Low PU: participants had limited exposure to AI tools, reducing their ability to perceive usefulness based on direct experience.
- Low PEOU: lack of training and infrastructure contributed to perceptions that AI would be difficult to use.

This aligns with Luo and Qin's (2021) argument that AI readiness in academic libraries depends heavily on institutional capacity and digital infrastructure.

## Theme 2: Perceived benefits of AI integration in library and information services

Despite limited implementation, participants expressed strong beliefs about the potential usefulness of AI in improving library operations. These perceptions align with the Perceived Usefulness (PU) construct of TAM.

Concerning the benefits of AI integration in libraries, the research found out that AI integration could lead to improved services and efficient library management. It was also discovered that use of AI-based tools in library routine tasks like categorizing and sorting books spared librarians adequate time to focus on user engagement initiatives like training of library users on the use of new technologies, which in turn improved efficiency and user satisfaction.

When one of the librarians was asked during the interview session about the benefits of AI integration in teachers’ college libraries, he said:

*"Because of the current limited integration of AI in libraries, we do not have much benefit. When AI is fully integrated in Libraries it will improve service delivery and operational efficiency. AI-based tools can be used to automate and perform routine library tasks leaving us with adequate time to focus on user engagement activities. AI and ML tools can be used to preserve library materials like books, documents and manuscripts by digitizing them."*

Buttressing the same viewpoint, the ICT expert who was also interviewed said:

*"One of the key benefits of AI integration in libraries is automation of routine tasks. Tasks previously requiring library personnel can perfectly be carried out by AI-based tools. This can help to improve the services offered to patrons. This in turn will increase user satisfaction."*

Library staff emphasized that AI could automate repetitive tasks such as cataloguing, classification, and circulation management. This aligns with global findings that AI enhances efficiency and reduces manual workload (Ali et al., 2021; Cox et al., 2019; Stafford, 2021).

The benefits of AI and ML integration in libraries were also discussed during the Focus Group Discussions with the students. A participant in Focus Group Discussion 1 emphasized that:

*"AI integration in the library has the benefit of improving efficiency through automation of library tasks which result in improved access to information. Use of chat-bots to respond to user queries helps to improves access to library information."*

Students highlighted the potential for AI-powered recommender systems and intelligent search tools to improve access to relevant materials. Another participant from the same Focus Group Discussion also said:

*"AI integration promotes personalised user experiences. Library users will have a personalised experience with their personal preferences being met. AI algorithms can be used to analyse user borrowing history and search queries in order to create personalized book recommendations. This will improve user satisfaction."*

Participants across all groups valued the possibility of 24/7 AI-powered chatbots to support users outside normal working hours. A participant from Focus Group Discussion 3 supported this idea when she said:

*"AI integration in libraries help to provide users with convenient services outside the regular working hours. AI powered Chat-bots provide 24/7 services to the clients in a timely manner."*

The observation done by the researcher showed the major benefits of AI integration as automation of the routine library tasks like categorizing and sorting of books.

Responses given by participants about the benefits of AI integration are in full support of the view by Asemi and Asemi (2018) who reiterated that AI algorithms can be used to analyse user borrowing history, ratings and search queries to create personalized book recommendations. Akinyemi (2023) also emphasised that AI powered systems can recommend books, articles and other relevant resources to library patrons which in turn enhances research.

Mogali (2015) in support of the above sentiments also explained that AI powered Chat-bots answer frequent user queries, which help to free librarians of their hectic activities of responding to user queries. This will spare librarians enough time to attend to more in-depth research assistance to users resulting in increased user satisfaction.

Supporting the same idea on the benefits of AI integration in libraries, Balasubramanian and Tamilselvan (2023) assert that AI algorithms can help to preserve library materials such as books, documents, and manuscripts by digitizing them which can help to reduce the risk of damage or loss to such physical materials and make them more easily and widely accessible to users.

### Interpretation through TAM

These perceived benefits reflect high PU, even in the absence of actual AI systems. Participants believed AI could enhance efficiency, accuracy, and user satisfaction which is consistent with findings from Fernandez (2023) and Yoon (2022). The responses from the research participants clearly show that AI integration in libraries has a lot of benefits including personalised recommendation systems, improved efficiency and user satisfaction. The responses given by the research participants agree with the TAM theoretical framework, which asserts that an individual’s decision to adopt and use the technology is mostly influenced by the perceived usefulness of the technology.

## Theme 3: Constraints and challenges affecting AI adoption

Participants identified several constraints that significantly shape the Perceived Ease of Use (PEOU) and feasibility of AI integration. With regards to constraints in the integration of AI in libraries, the research discovered ethical issues, resource constraints, lack of skills, inadequate data, and infrastructure challenges as major hindrances.

### Inadequate infrastructure

ICT experts emphasized unreliable internet connectivity, outdated computers, and insufficient server capacity. One ICT expert noted:

*"Our infrastructure cannot support advanced AI tools. Even Koha struggles during peak hours."*

When asked about the constraints in AI integration in libraries, one of the IT experts said:

*"Major constraints in AI integration in libraries include resource limitations and lack of skills. For AI and ML to be smoothly integrated there is need to have robust hardware and software supporting infrastructure. Library staff and patrons also need to be acquainted with relevant skills in order to be able to use AI-based tools. Without adequate infrastructure and required skills AI integration will remain a dream."*

### Limited funding

All stakeholder groups cited financial constraints as a major barrier. In support of this, one of the library staff asked during the interview had this to say:

*"The major limitation we face as libraries is lack of resources. AI-based tools require robust infrastructure to smoothly run and lack of funding is a major blow. Apart from resource challenges we also face skills deficit challenges. Library staff and patrons do not have adequate skills to work with AI and ML technologies."*

### Skills gaps and training needs

Library staff expressed limited confidence in their ability to use AI tools without extensive training. This aligns with global findings that digital literacy is a key determinant of PEOU (Marikyan and Papagiannidis, 2024). One respondent from the Focus Group Discussions said:

*"Every new technology requires good infrastructure and skills to be smoothly integrated. The major challenge being faced by libraries is that of inadequate infrastructure and lack of skills on both librarians and patrons."*

### Data availability and quality

Concurring with the above mentioned view, another IT expert also said:

*"Another big challenge faced by libraries in their efforts to integrate AI-based tools is the unavailability of adequate data. AI and ML algorithms depend on large datasets for them to be effective. Such large datasets are not always available in libraries."*

### Ethical and governance concerns

Participants raised concerns about data privacy, surveillance, and the potential misuse of user data—issues highlighted in global AI governance frameworks [United Nations Educational, Scientific and Cultural Organization (UNESCO), 2021; OECD, 2019]. The same IT expert noted:

*"Another constraint hindering AI integration in libraries is the issue of ethical considerations. Since AI is a recent technology, majority of institutions and governments do not have policies and frameworks to govern the use of these technologies."*

### Digital inequality

Students noted that inconsistent access to devices and internet connectivity limited their ability to benefit from AI-enabled services. Another Focus Group Discussion participant also reiterated that:

*"Lack of AI and ML skills on the part of library patrons is also hindering the integration of these technologies. There is need for adequate training of all library users in order to have smooth integration of the technologies."*

This reflects broader digital inequality patterns documented by Noble (2018) and Eubanks (2018).

When the researcher conducted observations on constraints in the integration of AI, the major challenges discovered were inadequate infrastructure, lack of skills among library staff and patrons, as well as issues to do with data quantity and quality requirements. The researcher also discovered connectivity issues as another constraint in AI integration. The colleges observed did not have enough internet bandwidth which can smoothly support AI integration.

Participants’ sentiments on constraints in the integration of AI in libraries were in line with what previous researchers discovered. For example, Mupaikwa (2024) in his research on “The application of Artificial Intelligence and Machine Learning in Academic Libraries in Zimbabwe” explained that there has been limited adoption of AI in libraries due to a number of challenges including technological, organisational, human as well as environmental. Massis (2018) also carried out research to examine artificial intelligence (AI) and its potential relationship to the library. The research unearthed some concerns that AI technologies could disrupt the library’s operations and services if not properly integrated. In support of this, Wu et al. (2015) carried out research on application of artificial intelligence (AI) and Machine learning (ML) technologies in CiteSeerX which is a digital library search engine. Concerning the challenges of AI and ML integration, the researchers highlighted the cost and scalability issues of integrating AI into already existing systems like CiteSeerX from scratch.

### Interpretation through TAM

These constraints significantly reduce PEOU, thereby lowering the likelihood of adoption, even when PU is high. Lack of skills as a major constraint is also supported by the TAM theoretical framework which emphasises that one of the critical drivers of technology adoption is perceived ease of use. When users perceive the technology as easy, they are bound to adopt and accept the technology. In this case, librarians may not find it easy to integrate AI due to lack of skills and training.

## Theme 4: Perceived application areas for AI in LIS

Concerning application areas of AI and ML in libraries, this current research discovered personalized recommendation systems, reference service applications, enhanced information retrieval through NLP (Konyushkova et al., 2020), automated cataloguing and classification, digital preservation and archiving, and intelligent research assistance as the major applications where AI and ML can be employed in libraries.

Cementing this claim, during the interview sessions librarians were asked about the library areas and tasks where AI and ML can be put to task. One of the librarians said:

*"When we fully integrate AI and ML in our libraries, recommendation services offered to patrons will become more personal. AI and ML algorithms will help to have personalized recommender systems where unique users will have their personalized recommendation of books, articles and other resources tailored to their unique needs and interests. AI and ML integration will also improve the reference service applications."*

Answering the same question on application areas of AI and ML in libraries, another library staff also said:

*"AI and ML algorithms can also be used in the archiving and digital preservation of physical copies. Machine learning algorithms can assist in automated content migration and format conversion and help to convert hard copies into digital format that is easy to preserve."*

When IT experts were interviewed, they subscribed to the application areas highlighted above. One of the IT experts had this to say:

*"Personalised recommendation systems, automated cataloguing and classification as well as enhanced information retrieval through the use of Natural Language Processing are the key benefits of AI and ML integration in libraries."*

Another IT expert also said:

*"AI integration also helps to improve efficiency in the library management process. AI-powered systems can be used to track and locate physical materials within the library which helps to improve the loan and return processes, resulting in a more engaging user experience."*

Participants in Focus Group Discussions were also asked about the application areas of AI and ML in libraries. These participants indicated that they were not aware of the many application areas. However, they emphasised more on improved reference service as the major application area of AI and ML in the libraries. One of the participants asserted that:

*"AI and ML algorithms can be used to develop Chatbots. AI-powered Chatbots offer users accessible and convenient services outside of regular library hours by responding to their inquiries in a timely and accurate manner. Artificial intelligence powered Chatbots can also be used as intelligent research assistants."*

Another Focus Group Discussion participant also said:

*"AI integration in libraries can help to come up with intelligent research assistants. Machine learning algorithms can help researchers in topic modelling and even to analyse large data sets to identify hidden thematic structures (Broman, 2020). This can help to establish topic-based browsing."*

Responses given by the participants were all in agreement with the findings of previous researchers on application areas of AI and ML in libraries. Asemi and Asemi (2018) explained that AI and ML algorithms have widely been applied in the library for recommendation systems. Hybrid recommender systems have been proposed to support patron‑driven acquisition and weeding (Rhanoui et al., 2020). The researchers explained that Artificial Intelligence algorithms are used to analyse user borrowing history, user search queries as well as user ratings to create personalized book recommendations. Akinyemi (2023) concurred by stating that AI powered systems can recommend relevant books, articles, or other resources based on a user’s previous borrowing or reading history. This personalization helps users find materials they might not have imagined and this improves the user experience in libraries.

In the same vein, Abayomi et al. (2020) reiterated that AI uses sophisticated algorithms to suggest relevant materials, identify authentic and credible sources and also to summarise complex information for library users which in turn helps to improve user research capabilities. According to Huang et al. (2020), effective recommender systems are using ML algorithms to analyse user borrowing history, search queries and ratings to generate personalized books and resource recommendations. This helps to have recommendations that cater for individual interests, fostering a deeper exploration of library collections.

McCallum et al. (2000) assert that another area dominated by machine learning in libraries is cataloguing and classification. Machine learning algorithms can analyse the content of books and other resources to automatically assign subject based headings and categorize materials. This saves library personnel valuable time and improves resource collection organization.

On the application of AI and ML as Intelligent Research assistants, Beel and Breitinger (2009) found out that Machine Learning algorithms can be used to analyse citations used in academic papers to automatically suggest related resources. This helps to improve research workflows for both students and scholars.

Previous research also discovered another application area of AI and ML in libraries as digital preservation and archiving. According to Balasubramanian and Tamilselvan (2023), artificial intelligence can help to preserve library materials such as books, documents, and manuscripts by digitizing them. The researchers explained that AI and ML algorithms can also assist in automated content migration and format conversion, ensuring the long-term accessibility of valuable digital collections. AI can also assist in the repair and restoration of damaged or deteriorating materials so that they remain accessible and usable for future generations.

### Interpretation through TAM

These application areas reflect high PU, demonstrating that participants can envision meaningful uses for AI even in the absence of current implementation. Previous research findings and the responses from the current study have shown that major application areas of AI and ML in libraries include personalised recommendation systems, automated cataloguing and classification, digital preservation and archiving, intelligent research assistance, and enhanced information retrieval.

### Cross-case summary

While all three colleges shared similar constraints, subtle differences emerged:

- College A had slightly better connectivity but lacked skilled staff.
- College B had more ICT expertise but outdated hardware.
- College C had the weakest infrastructure and lowest perceived readiness.

These variations highlight the need for context-specific AI adoption strategies rather than a one-size-fits-all approach.

## Discussion

The purpose of this study was to explore the perceived benefits, constraints, and emerging application areas of Artificial Intelligence (AI) integration in Library and Information Services (LIS) within teachers’ colleges in Masvingo Province, Zimbabwe. Although AI adoption in the studied libraries remains minimal, the findings provide valuable insights into early-stage perceptions, institutional readiness, and the socio-technical conditions shaping AI acceptance in resource-constrained academic environments. This discussion interprets the findings through the Technology Acceptance Model (TAM), digital inequality theory, and global AI governance frameworks, while situating the results within broader Global South scholarship.

### Interpreting findings through the technology acceptance model (TAM)

#### Perceived usefulness (PU): high but aspirational

Across all stakeholder groups, participants expressed strong beliefs about the potential usefulness of AI in improving library operations. Librarians emphasized automation of routine tasks, ICT experts highlighted efficiency gains, and students envisioned enhanced search and recommendation capabilities. These perceptions align with global findings that AI can streamline cataloguing, improve information retrieval, and enhance user experience (Ali et al., 2021; Cox et al., 2019; Zhang and Zhao, 2023).

However, in this context, PU is aspirational rather than experiential. Participants’ expectations are shaped by theoretical knowledge, exposure to AI in other domains, and global narratives about AI’s transformative potential. This reinforces Luo and Qin's (2021) argument that in low-resource environments, PU often reflects anticipated benefits rather than direct interaction with AI systems.

#### Perceived ease of use (PEOU): low and constrained by structural realities

While PU was high, PEOU was significantly lower. Participants expressed concerns about limited digital skills, lack of training, unreliable connectivity, outdated hardware, as well as insufficient technical support. These constraints directly reduce PEOU, consistent with TAM’s assertion that perceived difficulty lowers adoption intention (Davis, 1989; Venkatesh and Davis, 2000). In this context, PEOU is shaped not by system design as TAM traditionally assumes but by structural and infrastructural barriers.

This finding supports critiques that TAM inadequately accounts for environmental constraints in developing countries (Musakali and Mutula, 2023). It also highlights the need to integrate TAM with broader socio-technical frameworks.

### Digital inequality as a determinant of AI readiness

Digital inequality theory provides a powerful lens for understanding the constraints identified in this study. Participants’ experiences reflect multiple layers of inequality:

- Technical inequality: unreliable internet, outdated computers
- Skills inequality: limited training for librarians and users
- Institutional inequality: insufficient funding and policy support
- Access inequality: inconsistent access to devices among students

These findings echo global concerns that AI adoption risks deepening existing inequalities when deployed in contexts lacking adequate infrastructure (Noble, 2018; Eubanks, 2018). In the studied colleges, digital inequality directly shapes both PU and PEOU, thereby influencing AI readiness.

This underscores the importance of addressing structural barriers and not just user perceptions when planning AI integration in Global South academic libraries.

### AI governance, ethics, and institutional readiness

Participants raised concerns about data privacy, surveillance, and ethical use of user information. These concerns align with global AI governance frameworks such as:

- UNESCO’s Recommendation on the Ethics of AI [United Nations Educational, Scientific and Cultural Organization (UNESCO), 2021]
- OECD AI Principles (OECD, 2019). High‑level national strategies, such as the US National Security Commission on AI (2021), emphasise the need for infrastructure and talent – resources that are scarce in the teachers’ colleges studied.

Both frameworks along with the US National Security Commission on AI (2021) emphasize transparency, accountability, fairness, and human oversight. National AI readiness reports, such as that of the UK House of Lords (2018), highlight the need for skills and infrastructure – challenges that are magnified in Global South contexts. The findings suggest that even in low-resource contexts, stakeholders are aware of ethical implications and expect institutions to adopt AI responsibly.

However, the absence of institutional AI policies or guidelines in the colleges studied indicates a gap between global governance principles and local implementation. This gap highlights the need for context-appropriate governance mechanisms that address both ethical concerns and infrastructural realities.

### Cross-stakeholder and cross-case insights

#### Cross-stakeholder differences

- Library staff focused on operational efficiency and workload reduction.
- ICT experts emphasized infrastructural limitations and technical feasibility.
- Students prioritized user experience, access, and personalization.

These differences reflect each group’s role within the institutional ecosystem and underscore the importance of multi-stakeholder engagement in AI planning.

#### Cross-case differences

Although all colleges faced similar constraints, subtle differences emerged:

- College A had better connectivity but lacked skilled staff.
- College B had stronger ICT capacity but outdated hardware.
- College C had the weakest infrastructure and lowest readiness.

These variations demonstrate that AI adoption strategies must be context-specific, even within the same region.

### Contribution to global south scholarship

This study contributes to the growing body of Global South LIS scholarship in several ways:

1. Contextual specificity: It provides institutional insights rather than broad national generalizations.
2. Multi-stakeholder perspective: It integrates views from librarians, ICT experts, and technologically literate students.
3. Theoretical integration: It combines TAM with digital inequality theory and AI governance frameworks.
4. Early-stage adoption focus: It examines perceptions in contexts where AI is emergent rather than fully implemented.
5. Conceptual advancement: It highlights the need for an AI readiness model tailored to resource-constrained academic libraries.

### Implications for policy, practice, and research

#### Policy implications

- Institutions should develop AI governance policies aligned with UNESCO and OECD principles.
- National bodies should support AI adoption through funding, infrastructure development, and capacity building.

#### Practical implications

- Training programs for librarians and ICT staff are essential to improve PEOU.
- Incremental adoption starting with low-cost AI tools may be more feasible in resource constrained institutions.
- Libraries should prioritize AI applications that directly enhance user experience.

#### Research implications

- Future studies should examine AI adoption longitudinally as infrastructure improves.
- Quantitative studies could complement these findings by measuring PU and PEOU.
- Comparative studies across different types of academic institutions would deepen understanding of contextual differences.

## Conclusion

The integration of AI in libraries presents a transformative opportunity to enhance information access, personalize user experiences, and optimize library operations. By embracing these technologies while addressing the associated challenges, libraries can ensure they remain vibrant hubs of knowledge and learning in this digital age. The future of Libraries lies in a strategic partnership between human expertise and the power of AI, fostering a more efficient, user-centric, and equitable information ecosystem.

### Summary of key insights

Although the integration of AI in libraries promises several benefits, it has emerged that the integration process is not an easy task. The findings from this current study have pointed to the fact that AI integration in teachers’ college libraries in Masvingo District is still very low. It can also be concluded that if AI is fully integrated, libraries will enjoy a number of benefits including automation of hectic and time-consuming tasks like cataloguing and classification, personalised recommendation systems, enhanced information retrieval, digital preservation and archiving, and improved user experience through chatbots and recommender systems.

The research can also conclude that AI integration in libraries is hindered mostly by resource constraints, lack of skills, inadequate infrastructure, poor connectivity, data unavailability, and ethical concerns. On application areas of AI and ML, it can be concluded that recommendation systems, reference services, digital preservation and archiving, automated cataloguing and classification, and intelligent research assistance are the major application areas for AI and ML integration in the library.

Furthermore, this study concludes that the Technology Acceptance Model (TAM) provides a valuable interpretive framework for understanding AI readiness in resource-constrained settings, but it must be complemented by broader socio-technical perspectives that account for structural barriers, digital inequality, and governance considerations. The interplay between perceived usefulness and perceived ease of use is significantly mediated by infrastructural realities, institutional capacity, and the availability of training and support.

### Theoretical contributions

This study makes several theoretical contributions:

1. Extends TAM by demonstrating how PU and PEOU are mediated by structural constraints in low-resource environments.
2. Integrates digital inequality theory to explain how infrastructural disparities shape technology acceptance.
3. Bridges AI governance and LIS scholarship, highlighting the importance of ethical and policy considerations in early-stage AI adoption.
4. Proposes a context-sensitive understanding of AI readiness, emphasizing the need for models tailored to Global South academic libraries.

### Methodological contributions

The study contributes methodologically by employing a qualitative descriptive design with phenomenological sensitivity, suitable for contexts where AI adoption is emergent. The study also contributes by using a multi-stakeholder sampling strategy that captures diverse perspectives from librarians, ICT experts, and technologically literate students. Methodological contribution was also done by demonstrating the value of triangulating interviews, focus groups, and observations to understand socio-technical phenomena in under-researched settings.

### Practical and institutional implications

#### Institutional capacity building

- Libraries should prioritize training programs to enhance digital literacy and AI competencies among staff.
- Incremental adoption, starting with low-cost AI tools such as chatbots or automated cataloguing, may be more feasible.

#### Infrastructure development

- Investment in reliable internet connectivity, updated hardware, and server capacity is essential for enabling AI integration.

#### Ethical and governance frameworks

- Institutions should develop AI governance policies aligned with UNESCO and OECD principles to guide responsible adoption.
- Data privacy, transparency, and user consent must be central to any AI deployment.

#### User-centered service innovation

AI tools should be introduced in ways that directly enhance user experience, such as recommender systems and intelligent search tools.

### Policy implications

At the national and sectoral levels:

- Policymakers should support AI adoption through funding mechanisms, capacity-building initiatives, and digital infrastructure development.
- Teacher-training institutions should be prioritized, given their role in shaping the digital competencies of future educators.

### Recommendations

With several constraints being faced by library staff in the integration of AI leading to lower adoption of these technologies, the study makes the following recommendations:

- A collaborative approach involving the government, libraries, academia, and the private sector is essential to promote AI integration in library information services.
- Pre-service and in-service training for library staff and patrons on AI and ML applications in libraries to improve their attitudes and perceptions towards these technologies in libraries.
- Libraries should invest in digital infrastructure, capacity building, and ethical guidelines for the successful integration of AI and ML technologies to support library functions.
- There is need for exchange programs between libraries and industries aimed at smoothening the integration of AI-based tools into the libraries.
- Government to fund AI integration in tertiary institution libraries in order for such libraries to keep pace with the emerging information requirements and technological advancements.
- Libraries should develop context-specific AI governance strategies that align with global frameworks such as UNESCO’s Recommendation on the Ethics of AI [United Nations Educational, Scientific and Cultural Organization (UNESCO), 2021] while addressing local realities and constraints.
- A phased approach to AI adoption should be implemented in low-resource academic libraries, starting with low-cost, high-impact applications before progressing to more complex implementations.

#### Further research

This paper provides a brief overview of the constraints and benefits of AI integration in Library and Information Services. The study opens several avenues for further inquiry:

1. Longitudinal studies to track changes in AI readiness as infrastructure improves.
2. Quantitative studies to measure PU, PEOU, and behavioural intention across larger samples.
3. Comparative studies across universities, polytechnics, and teachers’ colleges to identify institutional differences.
4. Action research to evaluate the implementation of specific AI tools in real-world library settings.
5. Further research is needed to explore the long-term implications of these technologies on areas like information security, the future of work in libraries, and the development of ethical frameworks for AI-powered information services.
6. Additionally, comparative studies across different types of academic institutions in the Global South would help to build a more comprehensive understanding of AI readiness and adoption patterns in resource-constrained contexts.

## Data Availability

The raw data supporting the conclusions of this article will be made available by the authors, without undue reservation.
